# Monitoring HIV infection in Minas Gerais state: 15-year assessment of adults living with HIV initiating Antiretroviral Therapy

**DOI:** 10.1590/0037-8682-0360-2020

**Published:** 2020-12-11

**Authors:** Cássia Cristina Pinto Mendicino, Gabriella Jomara da Silva, Letícia Penna Braga, Enrico Antônio Colosimo, Mark Drew Crosland Guimarães, Cristiane Aparecida Menezes de Pádua

**Affiliations:** 1 Universidade Federal de Minas Gerais, Faculdade de Farmácia, Programa de Pós-Graduação Stricto Sensu em Medicamentos e Assistência Farmacêutica, Belo Horizonte, MG, Brasil.; 2 Universidade Federal de Minas Gerais, Faculdade de Medicina, Programa de Pós-graduação Stricto Sensu em Infectologia e Medicina Tropical, Belo Horizonte, MG, Brasil.; 3 Fundação Oswaldo Cruz, Escola Nacional de Saúde Pública, Programa Stricto Sensu de Pós-graduação em Epidemiologia em Saúde Pública, Rio de Janeiro, RJ, Brasil.; 4 Universidade Federal de Minas Gerais, Instituto de Ciências Exatas, Departamento de Estatística, Belo Horizonte, MG, Brasil.; 5 Universidade Federal de Minas Gerais, Belo Horizonte, MG, Brasil.

**Keywords:** CD4 lymphocyte count, HIV-1, Highly active antiretroviral therapy, Viral load

## Abstract

**INTRODUCTION:**

The first Brazilian HIV treatment recommendation was put forward in 1996, resulting in 12 subsequent guidelines. Several changes were made regarding “when” and “how” to begin treatment. The latest guideline recommends immediate initiation of antiretroviral therapy (ART). This study aimed to describe the evolution of HIV treatment among people living with HIV (PLHIV) who initiated ART between 2004 and 2018 based on the national guideline recommendations concerning T-CD4+ and VL measurements.

**METHODS:**

A cross-sectional analysis of data of PLHIV aged >18 years, in Minas Gerais who received ART between 2004 and 2018 was conducted. Clinical, therapeutic, and demographic information were obtained from national healthcare databases. The study was divided into four periods: 2004-2007, 2008-2012, 2013-2016, and 2017-2018. Descriptive analyses were performed.

**RESULTS:**

A total of 60,618 PLHIV initiated ART (67% male and 48% aged 25-39 years), 36% of whom had CD4 counts at ART initiation and 51% documented VL after ART initiation. The median CD4 count ranged from 288 to 373 cells/µL. The median time to ART initiation decreased from 604 to 28 days and was lower among males (*p* <0.01). The median time from ART initiation to the first VL result decreased from 101 to 62 days over the study period, while the median VL after ART initiation ranged from 2.3 to 1.7 log_10_ copies/ml.

**CONCLUSIONS:**

Although our results demonstrated that most recommendations were followed, there seemed to be little impact on CD4 counts and VL testing. This may result in an inadequate evaluation of ART effectiveness.

## INTRODUCTION

The primary goal of antiretroviral therapy (ART) is to suppress the viral load (VL) to undetectable levels in order to restore the immunologic response, to reduce opportunistic infections, and to improve survival^1^. In the general population, ART is associated not only with a substantial decrease in the probability of HIV transmission due to the reduction of VL but also with an improvement in the health of people living with HIV (PLHIV) due to an increase in T-CD4+ lymphocyte counts (T-CD4+)^2^. 

ART was introduced in Brazil at the beginning of the 1990s^3^. Since then, national guidelines based on international recommendations have been formulated by the government to guide treatment. The first guideline was introduced in 1996, resulting in the current 12 guidelines^4^ ^-^ ^15^. When new guidelines were introduced, recommendations were updated, especially concerning “when” and “how” to begin treatment. The key goal was to assess the benefits and risks beginning from the early phase of treatment. Patients were not to be exposed to the long-term adverse effects of ART or to the possibility of development of drug resistance. Drug resistance could lead to virologic failure and the achievement of poor clinical outcomes^16^. ART was not considered an emergency. Therefore, the postponement of ART initiation was justified to keep patients healthy^5^ ^-^ ^7^. 

The first treatment of an AIDS case based on the Centers of Diseases Control and Prevention (CDC) criteria took place in Brazil in 1987. However, it was based on specific and definitive diagnosis methods that required complex and sophisticated exams, which are not feasible in many countries^17^. In 1992, the T-CD4+ count was introduced as a marker of immunosuppression, resulting in a more accurate classification of AIDS cases^17^. In this way, the timing of ART initiation was not only based on clinical criteria but also on the T-CD4+ count^5^ ^-^ ^7^. Determining the best cut-off value for T-CD4+ for beginning treatment also changed over time: from T-CD4+ counts < 200 cells/µl to any level, as is the currently adopted guideline.

From 2004 to 2007, Brazilian guidelines recommended initiating ART for all patients who matched the clinical disease criteria and/or T-CD4+ count ≤ 200 cells/µl. The combination of zidovudine/lamivudine (ZDV/3TC) plus efavirenz (EFV) was chosen as the first-line regimen^10^ ^,^ ^11^. In 2008, the guideline’s recommendations were to increase the T-CD4+ count cut-off to 350 cells/µl^12^. After 2013, evidences indicated that early treatment decreased HIV transmission^18^. Thus, either T-CD4+ counts ≤ 500 cells/µl or VL >100,000 copies/ml were the laboratory criteria to initiate treatment. In addition, ART initiation based on tenofovir/lamivudine (TDF/3TC) plus EFV became the first-line regimen^13^ ^,^ ^14^. Finally, after 2017, the national guidelines have recommended the immediate initiation of ART to all PLHIV regardless of the T-CD4+ count or VL. Moreover, the EFV-based first-line regimens have been replaced with dolutegravir (DTG)-based first-line regimens (Table 1)^15^. 

**TABLE 1:** Recommendations of ART initiation for asymptomatic patients from Brazilian guidelines between 2004 and 2018.

	Cut-off value		
	VL	T-CD4 count+	
Year	(copies/ml)	(cells/µl)	First-line regimen to ART initiation
2004 - 2007	not considered	≤200	ZDV + 3TC + EFV
			
2008 - 2012	not considered	≤350	ZDV + 3TC + EFV
			
2013 - 2016	>100,000	≤500	TDF + 3TC + EFV
			
2017 - 2018	immediate ART initiation		TDF + 3TC + DTG
		

**VL:** viral load; **T-CD4+:** T-CD4+ lymphocyte count; **ZDV:** zidovudine; **3TC:** lamivudine; **EFV:** Efavirenz; **TDF:** tenofovir; **3TC:** lamivudine; **DTG:** dolutegravir.

All these changes in the recommendations regarding ART initiation have contributed to a potential positive impact on Brazilian HIV indicators. Between 2012 and 2018, the proportion of PLHIV maintained in public HIV healthcare services and on ART increased from 55% to 71% and from 44% to 66%, respectively^19^. A reduction in AIDS-related mortality rate (per 100.000 inhabitants) in Brazil has been observed since 2004: mean mortality rate, 6.0 (2004-2007), 5.7 (2008-2012), 5.5 (2013-2016), and 4.6 (2017-2018: preliminary data). However, in spite of the updated recommendations on a higher T-CD4+ threshold over the years, the mean incidences of AIDS cases (per 100.000 inhabitants) has not been reduced as expected: 20.4 (2004-2007); 21.6 (2008-2012); 20.2(2013-2016); 18.1 (2017-2018: preliminary data), respectively^20^ ^-^ ^23^.

Despite the immediate treatment after HIV diagnosis, measurements of T-CD4+ counts and VL continue to play an important role in HIV monitoring. Current guidelines worldwide have recommended measurement of the T-CD4+ count prior to initiation of ART to provide information on the overall immune function with a focus on early treatment. VL has been recommended two to eight weeks after ART initiation to monitor the effectiveness of ART with a focus on viral suppression^24^ ^-^ ^25^.

We hypothesized that the measurements of T-CD4+ counts before ART initiation and VL after ART initiation have improved over a 15-year period in the state of Minas Gerais, Brazil. Minas Gerais, located in the southeastern region of Brazil, is a large state (approximately 21 million inhabitants)^26^, where 3,418 HIV-diagnoses were recorded in 2018. This accounted for approximately 8% of Brazilian HIV-notifications^23^. The objective of this study was to describe the evolution of HIV treatment among adult patients (18 years old and above) living in Minas Gerais, that initiated ART from 2004 to 2018 based on national guidelines regarding T-CD4+ and VL measurements.

## METHODS

### Study design and population

We performed cross-sectional analyses of PLHIV data in the state of Minas Gerais, Brazil. All HIV-infected individuals (aged 18 years or older) who initiated ART between January 2004 and December 2018 were eligible for the analysis. The study was approved by an appropriate Ethics Research Committee.

### Data source and variables

Data on clinical/therapeutic (VL measurement, T-CD4+ count, ART use) and demographics (age, sex) variables were obtained from two national healthcare databases of the public Brazilian Unified Health System (SUS): Medication Logistics Control System (SICLOM) and Laboratory Tests Control System (SISCEL). Interaction with these databases is required for ART dispensing and obtaining laboratory results (VL and T-CD4+ count measurements) nationwide. The health databases were linked through a deterministic and probabilistic deduplication of records based on the patient’s full name, sex, date of birth, and city of residence. This created a common identifier in both the SISCEL and SICLOM systems. Data management, programming, and analysis were performed using MySQL, Pareia, and R software systems^27^ ^,^ ^28^. The matching identification number for each patient allowed the linkage of the databases and formed a single database with all data processed through the Statistical Analysis System (SAS, version 9.4).

For analysis, we adopted four study periods according to the main changes in the recommendations of Brazilian guidelines concerning the T-CD4+ count cut-off for ART initiation: Period I, between 2004 and 2007; Period II, between 2008 and 2012; Period III, between 2013 and 2016; and Period IV, between 2017 and 2018 (Table 1). Age was calculated based on the date of ART initiation, and the median age was estimated and categorized into groups: 18-24, 25-39, 40-49, 50-59, and >60 years. The date of ART initiation was based on the first record of ART dispensing, verifying that no other prescription was recorded at least in the previous three years. T-CD4+ counts comprised the last result preceded the date of ART initiation by 90 days^24^. This was categorized in accordance with the T-CD4+ count cut-offs of each period: Period I, <200 cells/µl; Period II, >200-349 cells/µl; Period III, 350-499 cells/µl and Period IV, >500 cells/µl. VL comprised the first result after ART initiation and was considered as a log_10_ value and was characterized according to the detection limits at the designated time period: undetectable VL, < 2.6 log_10_ copies/ml (from 2004 to 2009) or <1.7 log_10_ copies/ml (from 2010 to 2018); detectable VL, from > 2.6 log_10_ copies/ml (from 2004 to 2009) or > 1.7 log_10_ copies/ml (from 2010 to 2018) to < 5.0 log_10_ copies/ml (from 2004 to 2018); and very high VL, >5.0 log_10_ copies/ml (from 2004 to 2018)^29^.

Additionally, the date of the first T-CD4+ count prior to ART initiation was considered to estimate the median time to ART initiation^30^. The time to ART initiation was categorized according to the T-CD4+ count cut-offs of each period. Similarly, the median time to the first VL measurement after ART initiation was calculated and stratified into the VL categories.

### Data analysis

Descriptive analyses were performed for each period. Measures of central tendency (median), measures of dispersion (interquartile [IQR]), and absolute and relative frequencies were calculated for selected variables. The Kruskal-Wallis test was used to compare the continuous variables, and multiple comparisons were based on the Bonferroni method. Data management and data analyses were performed using the Statistical Analysis System (SAS, version 9.4).

## RESULTS

Table 2 shows the descriptive analysis of 60,618 PLHIV initiating ART between 2004 and 2018 in the state of Minas Gerais, Brazil. The number/proportion of individuals increased in the first three periods: from 11,701 to 17,844 (52%) and from 17,844 to 19,716 (10%); however, it decreased from period III to IV, from 19,716 to 11,357 (42%). Overall, we noticed a greater proportion of men (67%) and individuals aged 25-39 years (48%). Over the study period, the median (IQR) age decreased from 40 (33-47) to 34 (28-43) years (*p*-value < 0.01). ART first-line regimens including ZDV /3TC plus EFV, or lopinavir (LPV), or nevirapine (NVP), were gradually changed to regimens based on TDF/3TC plus EFV or DTG. Among the study population, we noticed a large proportion of missing data due to the lack of routine exams. Overall, 64% (n= 38,535) of participants did not have T-CD4+ counts at ART initiation, ranging from 59% in period I to 61% in period IV. The proportion of individuals presenting with T-CD4+ counts of less than 200 cells/μl were 13%, 16%, 7%, and 12% in each period, respectively. The medians (IQR) of T-CD4+ baseline counts in periods I and II with 288(163-469) and 255(120-353) cells/μl were lower than those of periods III and IV with 373(205-520) and 349(151-553) cells/μl, respectively (*p* <0.01). Similarly, 49% (n= 29,554) of participants did not document VL measurements after ART initiation, and this proportion increased from 23% to 67%. The proportion of undetectable VL in periods III and IV ranged from 15% to 16%, respectively, while the median (IQR) VL decreased from 1.9 (1.4-2.8) to 1.7 (1.4-2.6) in the same periods (*p* <0.01) Table 2. 

**TABLE 2:** Characteristics of HIV individuals initiating antiretroviral treatment (2004-2018), Minas Gerais State, Brazil (N=60,618).

Variable	Category or	Period								Total	
	statistic	I		II		III		IV			
		n	%	n	%	n	%	n	%	n	%
											
Age (years)	18-24	334	3	1024	6	2313	12	1598	14	5269	9
	25-39	5092	43	8136	45	9926	50	5748	51	28902	48
	40-49	3977	34	5206	29	4183	21	2235	20	15601	25
	50-59	1699	15	2465	14	2321	12	1192	10	7677	13
	≥ 60	599	5	1013	6	973	5	584	5	3169	5
	Total	11,701	100	17,844	100	19,716	100	11,357	100	60,618	100
	Median (IQR)^a^	40 (33-47)		39 (31-47)		35 (28-45)		34 (28-43)		37 (29-45)	
											
Sex	Male	7232	62	10829	61	14058	71	8659	76	40769	67
	Female	4469	38	7024	39	5658	29	2698	24	19849	33
	Total	11,701	100	17,844	100	19,716	100	11,357	100	60,618	100
											
ART	ZDV+3TC+EFV	5035	43	9067	51	2332	12	108	01	16542	27
regimen	ZDV+3TC+LPV	1005	09	2516	14	1076	06	028	0.5	4625	08
	ZDV+3TC+NVP	892	08	680	04	238	01	023	0.5	1833	03
	TDF+3TC+EFV	204	01	2000	11	13460	68	1748	15	17412	29
	TDF+3TC+DTG	-	-	-	-	-	-	8450	74	8450	14
	Others	4565	39	3581	20	2610	13	1000	09	11756	19
	Total	11,701	100	17,844	100	19,716	100	11,357	100	60,618	100
											
T-CD4+ ^b^	<200	1525	13	2774	16	1376	7	1381	12	7056	12
	200-349	1377	12	2562	14	1223	6	847	7	6009	10
	350-499	832	7	1025	6	1492	8	865	8	4214	7
	≥500	1052	9	824	5	1566	8	1362	12	4804	8
	No measure ^b^	6915	59	10659	60	14059	71	6902	61	38535	64
	Total	11,701	100	17,844	100	19,716	100	11,357	100	60,618	100
	Median (IQR) ^a^	288 (163-469)		255 (120-353)		373 (205-520)		349 (151-553)		302 (153-471)	
											
Viral load ^c^,	Undetectable	6497	56	7160	40	2905	15	1772	16	18334	30
	Detectable	2197	19	3562	20	4020	20	1867	16	11646	19
	Very high	298	3	376	2	310	2	100	1	1084	2
	No measure ^c^	2709	23	6746	38	12481	63	7618	67	29554	49
	Total	11,701	100	17,844	100	19,716	100	11,357	100	60,618	100
	Median (IQR) ^a^	2.3 (2.3-2.9)		2.0 (1.4-2.5)		1.9 (1.4-2.8)		1.7 (1.4-2.6)		2.3 (1.4-2.7)	

Periods I: 2004-2007; II: 2008-2012; III: 2013-2016; IV: 2017-2018. ^a^ p-value < 0.01; ^b^ The last T-CD4+ count until 90 days before ART initiation; ^c^ The first viral load measurement after ART initiation. **IQR:** interquartile range; **ART:** antiretroviral therapy; **ZDV:** zidovudine; **3TC:** lamivudine; **EFV:** efavirenz; **LPV:** lopinavir; **NVP:** nevirapine; **TDF:** tenofovir; **3TC:** lamivudine; **DTG:** dolutegravir; **T-CD4+:** T-CD4+ lymphocyte count (cells/µL) ; **VL:** viral load (log_10_ copies/ml) undetectable: <2.6 (2001-2009) or <1.7 (2010-2018); detectable ≥ 2.6 and < 5.0 (2001-2009) or ≤ 1.7 and < 5.0 (2010-2018); very high: >5.0.

Table 3 presents the estimated time to ART initiation and the time to the first VL measurement after ART initiation stratified by cut-off exams and sex. Among 22,083 participants (36%) with T-CD4+ counts at ART initiation, the median time (IQR) to ART initiation decreased dramatically from 604 days (84-804) in the first period to 28 days (16-43) in the last period and was lower among males between periods I, II, and III (*p* < 0.01). Furthermore, the median time to ART initiation was lower for individuals presenting with lower T-CD4+ counts, except in the last period, in which there was no difference between the T-CD4+ category and time to ART initiation. Approximately 51% of participants (n=31,064) documented VL measurements after ART initiation. The median time from ART initiation to the first VL result was considerably reduced from 101 days in the first period to 62 days in the last period (*p* <0.01), with a notable difference between the undectable and very high VL in the last period (96 days vs. 8 days). Overall, there was no difference between females and males in the time to the first VL measurement after ART initiation.

**TABLE 3:** Time to ART initiation and time to the first viral load measurement after ART initiation, stratified by routine exams and gender, of HIV individuals who initiated treatment between 2004 and 2018, Minas Gerais State, Brazil.

			Period									
Time to,	Variable	Categories	I		II		III		IV		Total	
days			Median	QR	Median	IQR	Median	IQR	Median	IQR	Median	IQR
ART	T-CD4+,	≤ 200	75	28-629	35	18-62	35	21-62	25	14-38	35	19-71
Initiation ^a^	cells/µl	200-349	528	178-793	168	42-582	50	28-348	28	18-41	87	34-623
		350-499	742	524-849	400	63-911	82	35-727	28	17-45	180	34-769
		≥500	755	589-849	776	44-1330	68	31-548	30	19-52	85	29-792
												
	Gender	Male	589	81-793	58	28-462	49	28-264	28	16-43	50	25-475
		Female	622	89-813	82	31-706	67	29-777	28	15-48	82	29-752
												
		Total	604	84-804	66	29-557	52	28-391	28	16-43	57	27-594
		Number of participants ^b^	4,786		7,185		5,657		4,455		22,083	
												
												
First	Viral load	undetectable	100	54-171	97	60-146	158	91-441	96	55-183	104	61-176
viral load		Detectable	104	48-209	74	31-153	85	40-188	32	11-83	76	30-161
after ART		Very high	121	47-279	128	39-447	126	47-371	8	2-85	116	33-315
initiation												
	Gender	Male	104	54-186	93	54-154	112	60-291	63	23-139	96	49-179
		Female	98	49-175	89	47-144	113	55-334	56	19-122	92	45-168
												
		Total	101	52-182	91	50-151	112	58-302	62	21-134	94	48-175
		Number of participants ^c^	8,992		11,098		7,235		3,739		31,064	

Periods I: 2004-2007; II: 2008-2012; III: 2013-2016; IV: 2017-2018. ^a^ based on the time between the date of the first T-CD4+ until the date of first ART prescription; ^b^ total of individuals having T-CD4+ exams until 90 days before ART initiation, ^c^ total of individuals having viral load measurement after ART initiation. **IQR:** interquartile range; **VL:** viral load (log_10_ copies/ml); **undetectable:** ≤2.6 (2001-2009) or ≤1.7 (2010-2018); **detectable:** ≥ 2.6 and ≤ 5.0 (2001-2009) or ≥ 1.7 and ≤ 5.0 (2010-2018); very high: ≥ 5.0; **T-CD4+:** T-CD4+ lymphocytes count.

**FIGURE 1:**
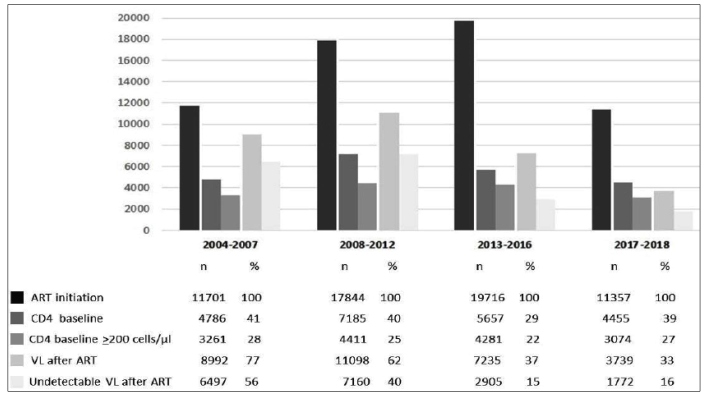
Proportion of CD4 baseline counts at ART initiation and viral load measurement after ART initiation of HIV individuals who initiated therapy between 2004 and 2018, Minas Gerais State, Brazil. **ART:** antiretroviral therapy; **CD4:** T-CD4+lymphocytes; **VL:** viral load (copies/mL log10) Undetectable: <2.6 (2001-2009) or <1.7 (2010-2018).

Figure 1 summarizes certain results presented with a focus on T-CD4+ and VL measurements. From 2004 to 2018, the increase of individuals who initiated ART was followed by a reduction in the proportion of both T-CD4+ counts and VL measurements. Additionally, over the time periods, there was a modest increase in the proportion of T-CD4+ >200 cells/μl and undetectable VL measurements. 

## DISCUSSION

Our study described selected results based on the updated recommendations of HIV monitoring from 2004 to 2018 in the state of Minas Gerais, Brazil. Certain findings were in accordance with the national guidelines adopted for each period.

According to the Brazilian guideline recommendations, ART first-line regimens based on ZDV were gradually switched to TDF as well as EFV to DTG. Meireles and collaborators presented similar frequencies of initial ART regimens in Brazilian HIV-infected individuals between 2014 and 2015 (n=76,950): TDF+3TC+EFV (75.6%), ZDV+3TC+EFV (5.3%), and ZDV+3TC+LPV (7.0%)^31^. Due to the decrease in the VL values, especially in the last period, this ART modification may have had a great impact on VL reduction. 

The national guidelines changed their recommendations to initiate ART earlier based on higher T-CD4+ thresholds. After 2017, these recommendations were changed to immediate ART initiation for all HIV-diagnosed individuals, regardless of the T-CD4+ count, with a preference for those with T-CD4+ counts below 350 cells/µl^15^. In fact, our results showed that the median time to ART initiation decreased and was prioritized in accordance with the T-CD4+ counts. A decreased time to ART initiation was associated with lower T-CD4+ counts. Our estimated findings in the state of Minas Gerais are similar to the national results. According to the Report of Clinical HIV Monitoring (2018), the median time between the first T-CD4+ count and ART initiation in 2009 and 2018 decreased markedly from 657 to 32 days in Brazil^30^.

National and international guidelines have recommended performing VL tests eight weeks after ART initiation to assess the virologic response and to identify virologic failure promptly^15^ ^,^ ^24^ ^,^ ^25^. We also noticed that the median time between ART initiation and the first VL measure is close to these recommendations, since it was reduced to just 62 days in the last period.

Despite all the advances previously described, the median T-CD4+ estimated counts showed a modest growth during the 15 years of HIV monitoring. Likewise, despite the improvements in time to ART initiation, the unchanged proportions of T-CD4+ counts <200 cells/μl over the period indicate a poor progress toward early HIV diagnosis. PLHIV with lower baseline T-CD4+ counts have advanced disease, poor immune system recuperation, and a higher risk of death, even after beginning ART. Similar to our results, Dias and collaborators evaluated 63,107 treatment-naïve HIV-infected individuals and estimated the mean of T-CD4+ counts varying from 348 in 2003 to 389 in 2009. A total of 52.3% of individuals presented with T-CD4+ counts below 350 cells/μl and 33.8% below 200 cell/μl in 2009^32^. Our findings are consistent with the small and slow reduction in the AIDS cases in Brazil^33^. 

We also observed a large proportion of HIV individuals on ART who had not completed routine tests. Even in the era of early ART and national/international guidelines recommend laboratory testing^15^ ^,^ ^24^ ^,^ ^25^, our results showed that 49% of individuals had no VL measures after ART initiation. This gap may compromise the “90-90-90” target to end the AIDS epidemic. The third “90” signifies that all people receiving ART should be virologically suppressed^34^. Furthermore, individuals without VL routine testing may impair the estimation of VL suppression after ART initiation. This measurement takes into account only the individuals with VL testing instead of the total number of individuals on ART^19^ ^,^ ^35^ ^,^ ^36^. As a VL test remains the key predictor of improvement with ART, low VL testing rates provide limited information about regarding the success of ART programs^37^. Additionally, VL testing after ART initiation improves adherence of ART^38^. 

Our study comprised a large period of time; thus, uncontrolled limitations inherent to the dynamic changes in the HIV epidemic must be addressed. In the early 2000s, the Health Ministry launched the SICLOM and SISCEL systems nationwide^39^. Thus, due to the implementation phase, the findings from the first years of our study require a more careful interpretation. The change in detection limits for undetectable VL in 2009 (from 2.6 log_10_ to 1.7 log_10_ copies/ml) impaired the comparison of the results from the first periods. Additionally, the number of VL tests conducted in the last months of 2018 is most likely underestimated due to the ending of the study period. As a result of these limitations, these VL results should be interpreted with caution. Second, the proportion of no values of both T-CD4+ count and VL testing may have affected the estimation of the median. Missing data can be partially explained due to a proportion of the population (27%) that received healthcare at private facilities^40^. T-CD4+ counts and VL data were not compulsorily recorded in the SUS databases. From December 2015, however, VL data from both public and private healthcare facilities have been required for ART dispensing^41^. 

Despite the recognized importance of T-CD4+ counts in evaluating the disease status at ART initiation, ART could have been initiated without performing a T-CD4+ count by the symptomatic patients who presented late for HIV/AIDS care (especially in the first years of the study period) or those who were clinically stable but did not perform T-CD4+ counts in view of the recommendation of early initiation of ART. Finally, the increasing demand for laboratory monitoring generated from earlier ART initiation can be a challenge for low- and middle-income countries in providing adequate coverage for both T-CD4+ counts and VL tests. In an African study to predict T-CD4+ count recovery, the number of participants was reduced by 83% (from 3,981,104 to 1,070,900) mainly due to the lack of T-CD4+ lymphocyte exams^42^. Similar results can be observed in Sub-Saharan Africa, where 11 million PLHIV are receiving ART. An estimated six million ART patients do not have access to VL testing^43^. 

There is scarce information regarding the availability of T-CD4+ counts and VL tests in Brazil. We believe that national studies on the frequency of routine exams might improve the availability of exams and indicate a more realistic scenario regarding immune status prior to ART initiation and VL suppression after ART initiation. Since 2014, data regarding new diagnoses of HIV infections have been incorporated into the national databases as a compulsory notification^44^. This action provided additional data on HIV surveillance including the date of HIV diagnosis, time of ART initiation, and immune status at the time of diagnosis. These steps would also likely improve adherence to routine exams. Despite all limitations, the large sample size and the wide time range improved the accuracy of our findings, providing a picture of the monitoring of people initiating ART in the state of Minas Gerais, Brazil.

Our study demonstrated that a majority of the recommendations regarding the national guidelines were followed over the study period. However, despite the national policy of universal access to ART (confirmed by a remarkable increase in ART availability noticed in our study), there remains a substantial gap between routine testing and ART initiation, which may lead to an inadequate evaluation of both immunological and virologic responses to ART. In addition, in the general population, it may contribute neither to a reduction in HIV transmission nor to the health of PLHIV.
